# Quantifying the short-term effects of air pollution on health in the presence of exposure measurement error: a simulation study of multi-pollutant model results

**DOI:** 10.1186/s12940-021-00757-4

**Published:** 2021-08-24

**Authors:** Dimitris Evangelopoulos, Klea Katsouyanni, Joel Schwartz, Heather Walton

**Affiliations:** 1 Environmental Research Group, School of Public Health, Imperial College London, Michael Uren Biomedical Engineering Hub, White City Campus, Wood Lane, W12 0BZ, London, UK; 2 NIHR HPRU in Environmental Exposures and Health, Imperial College London, London, UK; 3 National and Kapodistrian University of Athens, Medical School, Athens, Greece; 4 Department of Environmental Health, T.H. Chan School of Public Health, Harvard University, Boston, MA USA

**Keywords:** Air pollution, Measurement error, Mixture error, Effect transfer, Simulations, PM_2,5_, NO_2_

## Abstract

**Background:**

Most epidemiological studies estimate associations without considering exposure measurement error. While some studies have estimated the impact of error in single-exposure models we aimed to quantify the effect of measurement error in multi-exposure models, specifically in time-series analysis of PM_2.5_, NO_2_, and mortality using simulations, under various plausible scenarios for exposure errors. Measurement error in multi-exposure models can lead to effect transfer where the effect estimate is overestimated for the pollutant estimated with more error to the one estimated with less error. This complicates interpretation of the independent effects of different pollutants and thus the relative importance of reducing their concentrations in air pollution policy.

**Methods:**

Measurement error was defined as the difference between ambient concentrations and personal exposure from outdoor sources. Simulation inputs for error magnitude and variability were informed by the literature. Error-free exposures with their consequent health outcome and error-prone exposures of various error types (classical/Berkson) were generated. Bias was quantified as the relative difference in effect estimates of the error-free and error-prone exposures.

**Results:**

Mortality effect estimates were generally underestimated with greater bias observed when low ratios of the true exposure variance over the error variance were assumed (27.4% underestimation for NO_2_). Higher ratios resulted in smaller, but still substantial bias (up to 19% for both pollutants). Effect transfer was observed indicating that less precise measurements for one pollutant (NO_2_) yield more bias, while the co-pollutant (PM_2.5_) associations were found closer to the true. Interestingly, the sum of single-pollutant model effect estimates was found closer to the summed true associations than those from multi-pollutant models, due to cancelling out of confounding and measurement error bias.

**Conclusions:**

Our simulation study indicated an underestimation of true independent health effects of multiple exposures due to measurement error. Using error parameter information in future epidemiological studies should provide more accurate concentration-response functions.

**Supplementary Information:**

The online version contains supplementary material available at 10.1186/s12940-021-00757-4.

## Introduction

Air pollution is the major environmental factor affecting human health [1, 2]. Many publications report associations of morbidity and mortality outcomes with exposure to air pollutants, such as PM_2.5_, NO_2_ and O_3_ [3, 4]. It is important to assess the independent effects of each pollutant adjusting for potential confounding by other pollutants in the mixture, as policies to reduce emissions differ by targeted pollutant. Multi-pollutant models are the most commonly used method, and, if correctly specified, provide estimates of the independent effects of each exposure [5–7].

Most studies addressing the associations between pollutants and health, or performing health impact assessments (HIA), do not account for exposure measurement error (ME), although it is a well-known problem [8–11]. ME may lead to biased concentration-response functions (CRFs) for which the magnitude and type of bias is not assessed or corrected. While the methodology to correct for ME is expanding, there is a gap between theory and practice [12, 13]. To evaluate ME we must define the “gold standard” against which “proxy” measures are compared. For exposure to ambient concentrations, the most appropriate gold standard is individual exposure to ambient sources of the pollutant of interest. However, most epidemiological studies investigating ME in air pollution exposure, measure total personal exposure, to which indoor and personally-generated sources contribute substantially [14].

An appropriate framework describing the types of error for exposure variables has not been discussed thoroughly in the literature. Zeger et al. *(2000)* mention that “*classical and Berkson models represent two extremes of a continuum*”, so the proper model combines elements of each type [9]. Few studies have examined the impact of mixture error [15–17]. Previous studies examining the impact of ME in regression models either only used single-pollutant models or addressed only spatial heterogeneity of the pollutants and their errors or have made specific assumptions for their error definition using empirical data from certain locations which might not be generalisable [18–23]. Multi-pollutant models, in addition, provide unstable estimates because of the complex relationships between pollutants, both in space and time, and the differing degree and structure of their errors - especially the variability and correlation between errors.

In our study, we focus on multi-pollutant models using a mixture error model of classical and Berkson components based on the error decomposition of Zeger and colleagues (2000). The percentages of each type depend on the pollutant under investigation, i.e. fine particulate matter, PM_2.5_, or nitrogen dioxide, NO_2_. We used simulated data to generate a wide range of plausible scenarios of the pollutant errors under a mixture error model, and to quantify the real impact of ME on the multi-pollutant model effect estimates, specifically in time-series analysis.

## Methods

### Simulation set-up

We previously conducted a systematic review on the differences between ambient concentrations and personal exposures from outdoor origins to inform our simulation inputs and quantify the magnitude and variability of the pollutant errors [24]. In particular, we created a simulated daily time-series for error-free (A) and error-prone (C) exposures and a health outcome over a period of 4 years, under various scenarios for factors that drive ME bias (Fig. S1, Supplementary material). Our focus was only on the quantification of exposure measurement error bias, so we did not include some features of time-series that may introduce other forms of bias and need to be taken into account in real data analysis, such as autocorrelation, trends or confounders measured with error.

**Fig. 1:**
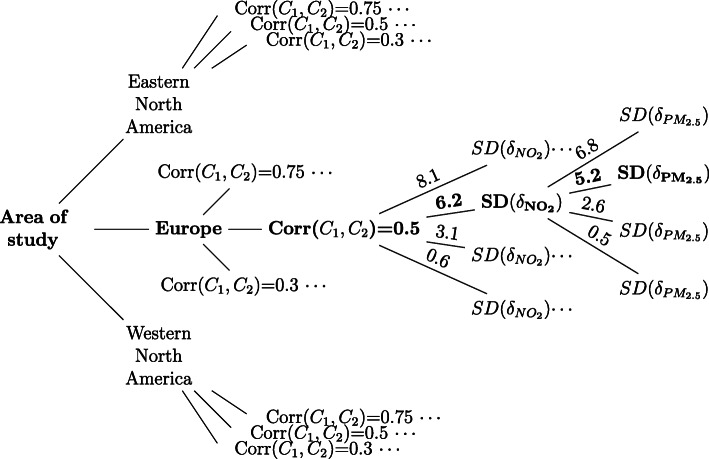
Diagram showing the construction of the 144 scenarios assumed in the analysis

Each scenario was created as a combination of key parameters driving ME bias, i.e. the correlation between the exposures, the error variability ($SD\left({\delta}_{P{M}_{2.5}}\right)$, $SD\left({\delta}_{NO_2}\right)$) and the true exposure variance/error variance ratio ($SD\left({A}_{P{M}_{2.5}}\right)/ SD\left({\delta}_{P{M}_{2.5}}\right), SD\left({A}_{NO_2}\right)/ SD\left({\delta}_{NO_2}\right)$) [23]. The latter was informed according to data from three different areas, for which we had adequate information [24]. In total, 144 scenarios were investigated, and their inputs are summarised in Figure 1. Due to space constraints, we expanded only one branch in the tree plot (in bold); this was assumed to be the core scenario compared with which all sensitivity analyses were performed. For each scenario, 1000 simulated datasets were generated.

Briefly, we assumed:

- Variance ratios: We created scenarios separately for Europe, Eastern and Western North America which correspond to different ratios of the true exposure variance over the error variance. Differences in the ratios between these regions can be attributed to various factors, such as the use of Diesel vehicles, air conditioning, and legislation [25].
- Correlation between the exposures: To the best of our knowledge, no data for the correlation between personal exposures from outdoor sources to PM_2.5_ and NO_2_ are reported in the literature. Thus, we assumed that it lies between published correlation coefficients of total personal exposures and correlations of the ambient measurements for the same pollutants [26–28]. A range of values was hypothesized to cover various plausible scenarios.
- Correlations between the errors: Because we did not have real data to estimate the relationship between these errors, an analytical approach for their covariance was applied using the pairwise associations of personal and ambient concentrations for NO_2_ and PM_2.5_ (eq. 2).
- Error structures: Little is known about the distributions and types of exposure ME. We assumed that the errors are additive on the logarithmic scale [29, 30]. Moreover, we assumed that a mixture of classical and Berkson error best describes the type of exposure misclassification [9]. However, scenarios of entirely classical and entirely Berkson error, alternative figures for the classical-Berkson ratio and multiplicative error were also assessed.

### Input data

The key input parameters are summarised in Table 1. We assumed different “true” exposures and error variability for each study area which resulted in different exposure variance over error variance ratios. Three correlation coefficients between the exposures (low = 0.25/moderate = 0.5/high = 0.75) and four values for the error variability for each pollutant were hypothesized. For example, for Europe (core scenario) we hypothesized moderate error variability (5.2 μg/m^3^ for PM_2.5_ and 6.2 ppb for NO_2_), very low (0.5 μg/m^3^ and 0.6 ppb respectively (0.1 × moderate)), low (2.6 μg/m^3^ and 3.1 ppb respectively (0.5 × moderate)), and high (6.8 μg/m^3^ and 8.1 ppb respectively (1.3 × moderate)). The 144 scenarios tested derived from all the possible combinations of these parameters.

**Table 1:** Simulation inputs for the assumed “true” exposures and the error variability of the “error-prone” exposures

Area of Study	“True” PM_2.5_ - Mean (SD) (μg/m^3^)	“True” NO_2_ - Mean (SD) (ppb)	PM_2.5_ Error - SD (μg/m^3^)	NO_2_ Error - SD (ppb)
Eastern North America	19.0 (8.6)	20.7 (11.6)	5.7	7.3
Europe (core scenario)	21.1 (10.9)	21.6 (8.9)	5.2	6.2
Western North America	18.7 (8.3)	22.4 (10.9)	5.6	7.3

### Generating "error-free" or "true" exposures (A):

We used a bivariate log-normal distribution to generate 1461 daily measurements of the “error-free” exposures for each scenario **s**, as follows:

$$ Lognormal\left({\boldsymbol{\mu}}_{\boldsymbol{s}},{\boldsymbol{\varSigma}}_{\boldsymbol{s}}\right),{\boldsymbol{\mu}}_{\boldsymbol{s}}=\left[\begin{array}{c}{\mu}_1\\ {}{\mu}_2\end{array}\right],{\boldsymbol{\varSigma}}_{\boldsymbol{s}}=\left[\begin{array}{cc}{\sigma}_1^2& {\sigma}_{1,2}\\ {}{\sigma}_{2,1}& {\sigma}_2^2\end{array}\right] $$

where ***μ***_***s***_ is the vector of pollutant means, ***Σ***_***s***_ the variance-covariance matrix and 1, 2 = NO_2_ and PM_2.5_, respectively. For example, for the European area, we had:

$$ {\boldsymbol{\mu}}_{\boldsymbol{eu}}=\left[\begin{array}{c}21.6\\ {}21.1\end{array}\right],{\boldsymbol{\varSigma}}_{\boldsymbol{eu}}=\left[\begin{array}{cc}{9.0}^2& 27.6\\ {}27.6& {10.9}^2\end{array}\right]. $$

### Generating “error-prone” or “apparent” exposures (C):

We considered a mixture of classical and Berkson ME additive on the logarithmic scale as the most appropriate model because the sources of error can originate from both classical error (omitting individuals’ mobility, the infiltration efficiency of the buildings, and instrument errors) and Berkson error from unincorporated spatial heterogeneity [9]. Various scenarios for the errors allowed evaluation of how the error type affects the health effect estimates, especially in two-pollutant models where the magnitude and direction of bias cannot easily be predicted in typical epidemiologic studies without specifically investigating ME.

#### Classical error

Apparent exposures of classical error were generated simply by adding random error to the assumed true exposures. For this type of error, we have:

1 $$ {C}_i^t={A}_i^t+{\delta}_i^t $$

where i is NO_2_ or PM_2.5_, t is day, t = 1,2, …, 1461, C and A are as defined above, and the errors, δ, of the pollutants were created from a bivariate normal distribution:

$$ {\delta}_i^t\mid {A}_i^t\sim N\left(\mu, \varSigma \right),\mu =\left[\begin{array}{c}0\\ {}0\end{array}\right],\varSigma =\left[\begin{array}{cc} Var\left({\delta}_{N{O}_2}^t\right)& Cov\left({\delta}_{N{O}_2}^t,{\delta}_{P{M}_{2.5}}^t\right)\\ {} Cov\left({\delta}_{N{O}_2}^t,{\delta}_{P{M}_{2.5}}^t\right)& Var\left({\delta}_{P{M}_{2.5}}^t\right)\end{array}\right] $$

The elements in the diagonal of **Σ** are the error variances as estimated from [24]. The covariance was estimated analytically:

2 $$ Cov\left({\delta}_{N{O}_2}^t,{\delta}_{P{M}_{2.5}}^t\right)= Cov\left({C}_{N{O}_2}^t-{A}_{N{O}_2}^t,{C}_{P{M}_{2.5}}^t-{A}_{P{M}_{2.5}}^t\right)= Cov\left({C}_{N{O}_2}^t,{C}_{P{M}_{2.5}}^t\right)- Cov\left({C}_{N{O}_2}^t,{A}_{P{M}_{2.5}}^t\right)- Cov\left({A}_{N{O}_2}^t,{C}_{P{M}_{2.5}}^t\right)+ Cov\left({A}_{N{O}_2}^t,{A}_{P{M}_{2.5}}^t\right) $$

There was no information in the literature for the associations between C and A, so we used covariances from studies of total personal exposure instead [26–28].

#### Berkson error

In general, a Berkson error model can be described as follows:

3 $$ {A}_i^t={C}_i^t+{\delta}_i^t, $$

We used a formula to generate Berkson error proposed by Carroll et al. (2006) [11]. Briefly, if C = A + δ (i.e. classical model) we know that the best linear predictor of A given C is (1-λ)∙E(A) + λ∙C, where $\lambda =\frac{\sigma_C^2}{\sigma_C^2+{\sigma}_{\delta}^2}$. Then, the following equation (4) results in the generation of a Berksonian-type error-prone exposure. For simplicity, we omitted the indicators i, t:

4 $$ C={\mu}_A+\left(A-{\mu}_A\right)\bullet \frac{\sigma_C^2}{\sigma_C^2+{\sigma}_{\delta}^2}+{\delta}^{\ast } $$

where ${\delta}^{\ast}\sim N\left(0,\frac{\sigma_C^2\bullet {\sigma}_{\delta}^2}{\sigma_C^2+{\sigma}_{\delta}^2}\right)$ and ${\sigma}_{\delta}^2$ is the error variance (Supplementary material).

#### Mixture error

Using the error decomposition from Zeger and colleagues [9], with data from other studies [31, 32], we quantified the error-prone variables of the mixture type. We estimated the error as 43% classical and 57% Berkson for PM_2.5_ and 33% classical and 67% Berkson for NO_2_ in the main analysis, while in sensitivity analyses we assumed increased percentages of classical error for both pollutants, i.e. either (55,45%) for PM_2.5_ and (45,55%) for NO_2_ or (70,30%) for PM_2.5_ and (60,40%) for NO_2_. As the hypothesized true exposures are log-normally distributed, we assumed additive error on the log-scale for the ME models based on previous studies, but also applied a multiplicative approach [29, 30].

The mixture model consists of the variables described in eqs. 1 and 2, along with a latent intermediate variable L, between A and C, that allows for mixtures of Berkson and classical error as described elsewhere [15, 33]. Briefly, the model is:

$$ A=L+{\delta}_b $$

$$ C=L+{\delta}_c $$

where δ_b_, δ_c_ denote Berkson-type and classical-type error (Supplementary material).

### Generating all-cause mortality

For each day t, the number of deaths Y_t_ was assumed to follow a Poisson distribution with overdispersion φ. The mean μ_t_ of the Poisson distribution was created to be dependent only on the simulated “true” concentrations of both pollutants on every particular day as follows:

$$ \log \left({\mu}_t\right)={\beta}_0+{\beta}_1\bullet {A}_{N{O}_2}^t+{\beta}_2\bullet {A}_{P{M}_{2.5}}^t $$

where ${A}_{N{O}_2}^t,{A}_{P{M}_{2.5}}^t$ are the daily concentrations for NO_2_ and PM_2.5_, respectively.

To simulate this Poisson over-dispersed data, we assumed that daily mortality followed a Negative Binomial distribution, NB (μ_t_,θ), where μ_t_ is the mean daily number of deaths, and θ = φ/(μ_t_-1). To get reasonable estimates for β_0_, φ we used real mortality and air pollution data during 2011–2014 in Greater London.

For the selection of β_1_, β_2_ we reviewed the literature for studies that reported associations between short-term exposure to NO_2_ and PM_2.5_ and all-cause mortality and derived their CRFs. We chose plausible CRF values from a recent meta-analysis of time series studies despite the fact that they are not adjusted for ME, i.e. a 0.6 and 0.54% increase in all-cause mortality per 10 units increase in NO_2_ and PM_2.5_ respectively (β_1_ = 0.0006, β_2_ = 0.00054) [34]. We also tested whether the results remain unchanged when the true coefficients were assumed to be half or twice the above percentage increases or when only one pollutant had a true health effect (β_i_ = 0 for the co-pollutant).

### Epidemiological analysis

In the context of this study, we are interested in the ME bias - quantified as the difference between the health effect estimates of error-free and error-prone exposures. The Poisson time-series model allowing for over-dispersion used in every iteration was:

$$ \log \left(E\left({Y}_t\right)\right)={\beta}_0+{\beta}_1\bullet {C}_{N{O}_2}^t+{\beta}_2\bullet {C}_{P{M}_{2.5}}^t $$

where Y_t_ is the death count for day t and ${C}_{N{O}_2}^t$, ${C}_{P{M}_{2.5}}^t$ the corresponding error-prone exposure based on every scenario. We also calculated coverage probability as the percentage of 95% confidence intervals that include the assumed true exposure-response association, and power as the percentage of statistically significant estimates at the 5% level.

## Results

We confirmed that the generated exposure variables had close to the expected mean values (Table 1) and the expected distributions for the classical, Berkson or mixed error types (Fig. S2, Supplementary Material).

Table 2 contains the average Poisson regression estimates across all scenarios by areas under investigation. Pure Berkson error model resulted in overestimation of the true mortality effect for all areas and pollutants by 1.5 to 8.2%, except for the NO_2_ coefficient for Europe (a decrease of 7.9%), the assumed area scenario with the lower exposure variance/error variance ratio and higher corresponding ratio for the co-exposure. Otherwise, the mortality effects were underestimated with greater bias observed in Europe for NO_2_ (27.4%, mixture model). Interestingly, the bias in North America was similar for both pollutants, around 15–19% for classical error and 9–13% for mixture (area scenarios with similar, and relatively low exposure variance/error variance ratios for both pollutants). In Europe, the bias was significantly increased for NO_2_ compared with PM_2.5_, 10.0 and 25.1% for classical and 1.3 and 27.4% for mixture error respectively. Coverage and power for both pollutants across all scenarios by area and error type were 51–60% and 60–80% respectively.

**Table 2:** Summary of the true and error-prone regression coefficients, their standard errors (SE) x 10^-4^ and relative bias from 144,000 simulated datasets on the impact of three error models (classical, Berkson and mixture) on 2-pollutant Poisson regression by area of study. Results presented for all scenarios (*N* = 48,000 in each row)

Exposure	CRFs^a^:	PM_2.5_: β_1_ = 5.4^a^					NO_2_: β_2_ = 6^a^				
	Area	${\hat{\boldsymbol{\beta}}}_{\mathbf{1}}$	(SE_**W**_)/(SE_**B**_)^**b**^	Bias (%)^**c**^	Coverage Probability (%)	Power (%)	${\hat{\boldsymbol{\beta}}}_{\mathbf{2}}$	(SE_**W**_)/(SE_**B**_)^**b**^	Bias (%)^**c**^	Coverage Probability (%)	Power (%)
True	Europe	5.37	(1.48)/(3.46)	–	–	–	6.00	(1.79)/(4.20)	–	–	–
	East NA	5.42	(1.88)/(4.41	–	–	–	5.98	(1.39)/(3.25)	–	–	–
	West NA	5.40	(1.95)/(4.58)	–	–	–	5.99	(1.48)/(3.45)	–	–	–
Classical	Europe	4.86	(1.36)/(3.27)	−10.0	55.3	76.0	4.49	(1.52)/(3.82)	−25.1	51.1	67.6
	East NA	4.58	(1.67)/(4.02)	−15.1	54.6	65.2	4.97	(1.25)/(3.09)	−17.2	53.9	80.4
	West NA	4.52	(1.72)/(4.17)	−16.3	54.3	63.7	4.85	(1.30)/(3.24)	−19.2	52.8	77.2
Berkson	Europe	5.84	(1.70)/(4.02)	+ 8.2	59.3	75.4	5.53	(2.46)/(5.89)	−7.9	58.8	62.2
	East NA	5.78	(2.73)/(6.43)	+ 7.1	59.3	62.2	6.17	(1.95)/(4.58)	+ 2.8	59.7	75.6
	West NA	5.62	(3.29)/(7.88)	+ 4.1	59.3	60.2	6.09	(2.50)/(5.87)	+ 1.5	59.2	71.1
Mixture^d^	Europe	5.33	(1.49)/(3.57)	−1.3	57.9	76.1	4.36	(2.00)/(5.10)	−27.4	55.0	60.8
	East NA	4.91	(1.91)/(4.61)	−9.1	57.1	63.2	5.37	(1.48)/(3.58)	−10.6	58.0	76.9
	West NA	4.84	(1.99)/(4.81)	−10.3	57.2	61.5	5.20	(1.58)/(3.85)	−13.3	57.1	72.8

^a^Concentration-response functions for generation of the health outcome

^b^ SE_W_:Within-simulations (or model-based) standard error, SE_B_:Between-simulations (or empirical) standard error

^c^ Relative bias = $$ \frac{\left({\hat{\boldsymbol{\beta}}}_{\boldsymbol{\iota}}-{\boldsymbol{\beta}}_{\boldsymbol{\iota}}\right)}{{\boldsymbol{\beta}}_{\boldsymbol{\iota}}} $$

^d^ (Classical,Berkson) percentages: (43,57%) for PM_2.5_, (33,67%) for NO_2_

**Fig. 2:**
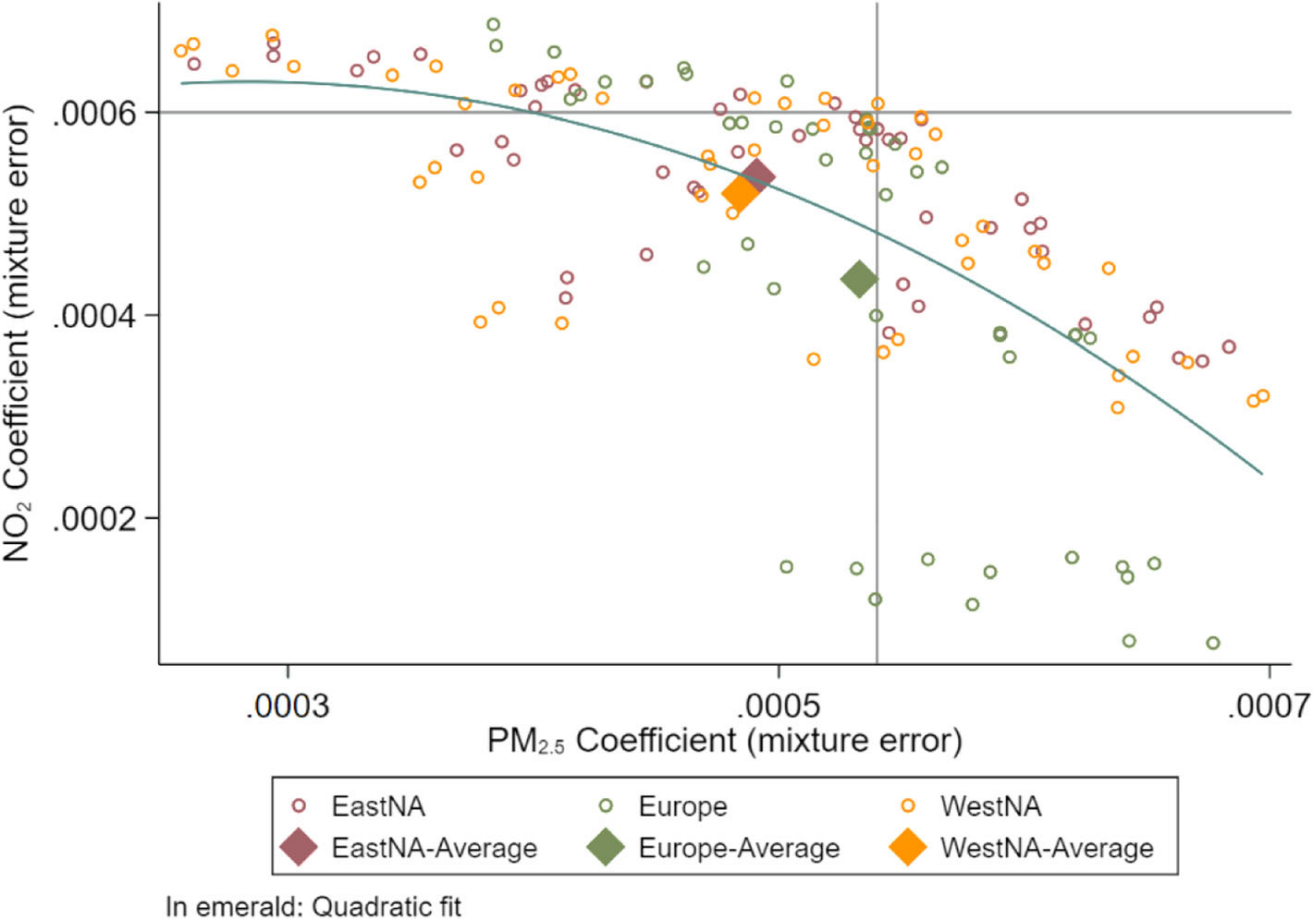
Scatter plot of the PM_2.5_ and NO_2_ Poisson regression coefficients by area of study when mixture error was assumed. In dots are the averages of 1000 simulated datasets across the same scenario. Diamonds show the averages of all the datasets assuming the same area of study. The two lines illustrate the assumed true mortality effect of the pollutants. Results presented for all 144 scenarios

The results of mixture error are illustrated in Fig. 2 showing the relationship between PM_2.5_ and NO_2_ coefficients by study area for all scenarios. No scenario resulted in overestimation of the effect of both pollutants (upper right quartile). Between pollutants, higher values of ${\hat{\beta}}_1$ tend to be associated with lower values of ${\hat{\beta}}_2$, in an approximate quadratic negative relationship. No clear pattern was observed by study area, except for a group of European results in the lower right of the graph (> 70% decrease in the NO_2_ effect estimate). NO_2_ coefficient attenuation is higher than in other scenarios because the hypothesized exposure variance/error variance ratio was lower compared to PM_2.5_.

**Fig. 3:**
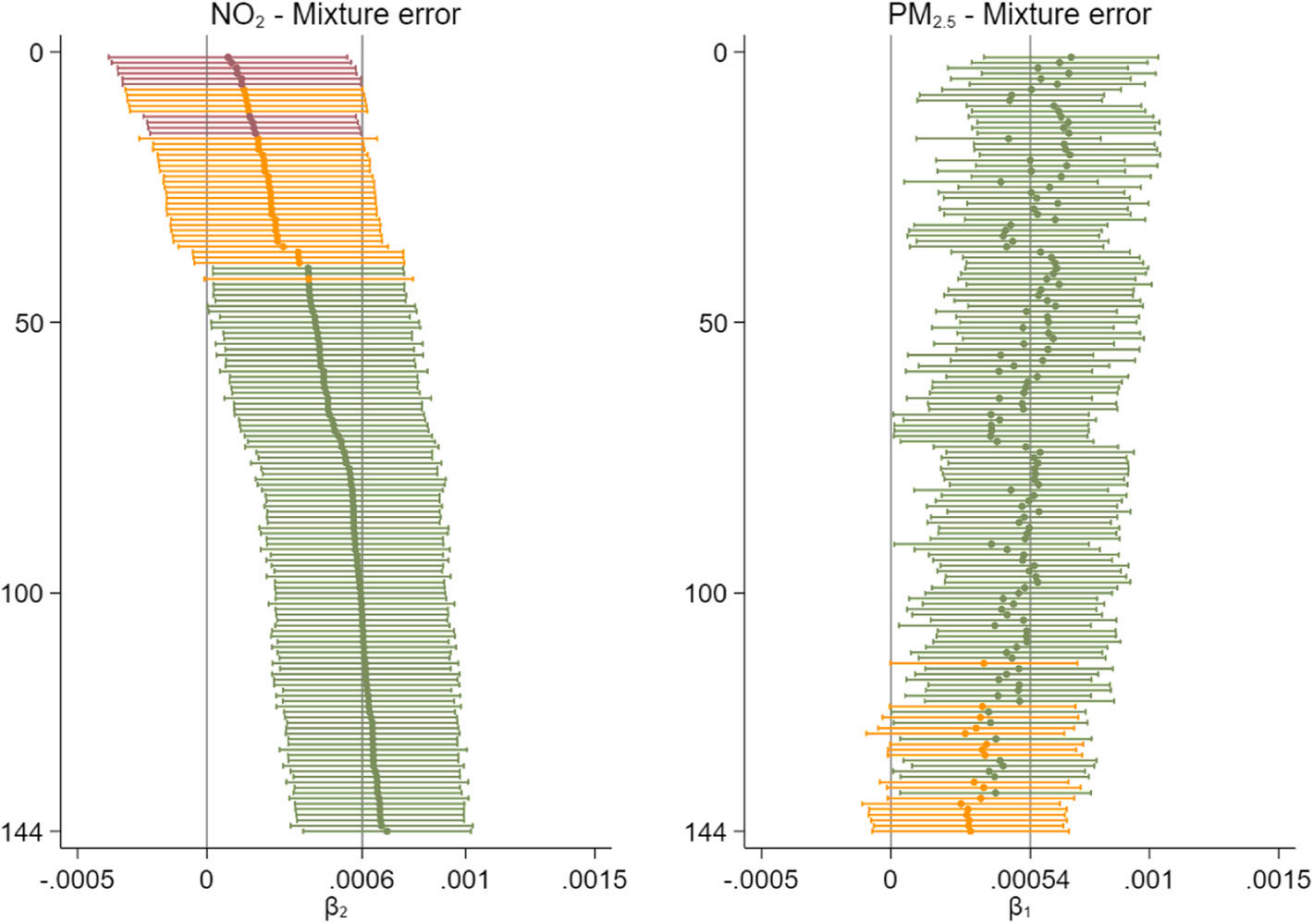
Plot for the comparison of PM_2.5_ and NO_2_ mortality estimates (with 95% CIs) for mixture error type (averages of 1000 simulated datasets across the same scenario, sorted by the NO_2_ regression coefficients). Results presented for all 144 scenarios. The vertical lines illustrate the assumed true mortality effect of the pollutants. In green: statistically significant estimates, in orange: not-statistically significant estimates, in red: significantly biased estimates

Figure 3 plots the mortality estimates for both pollutants sorted in increasing order of NO_2_ effect size (left). Effect transfer can clearly be observed either from NO_2_ to PM_2.5_ (upper part) or from PM_2.5_ to NO_2_ (lower part), but also, there are some scenarios where both coefficients were attenuated (middle part). Interestingly, in the middle and even at the top of the graph, there are scenarios in which the estimates for both pollutants are to the left of the true beta line i.e. attenuated. Nonetheless, large attenuations for both pollutant estimates did not occur simultaneously, as can be seen from the absence of points in the bottom left corner of Fig. 2.

Table 3 illustrates the model estimates with different assumed values for the correlation between the exposures and the error variances. Results from the most plausible error model are reported, i.e. mixture error. Effect transfer is clearly observed, as the effect estimates for one exposure are decreasing when its error variability is increasing (less precise exposure), while the co-exposure’s coefficients are increasing, and its error variability is kept fixed. For example, in the “moderate” correlation scenarios, keeping the PM_2.5_ error variability fixed at “low” (SD(${\delta}_{P{M}_{2.5}}$) = 2.9 μg/m^3^), the bias in the PM_2.5_ Poisson regression estimates range from − 3.7% in the “very low” NO_2_ error variability scenario to + 17.8% in the “high” scenario. On the contrary, the corresponding bias for NO_2_ varies from + 0.7% to − 50.0% (“very low” to “high” SD(${\delta}_{NO_2}$)). Bias in the effect estimates varies highly by assumed error structure from 26.1% overestimation to 44.0% underestimation of the true effect for PM_2.5_, and from + 12.1% to − 58.4% for NO_2_. The highest biases are observed in the most extreme scenarios, but in the more plausible “low”-“moderate” error variability scenarios, bias is not negligible (values up to approximately 30% towards the null). Correlation between the exposures does not seem to be a driving factor for the bias.

**Table 3:** Summary of the regression coefficients, their standard errors (SE)(×10^− 4^) and relative bias of 144,000 simulated datasets on the impact of mixture error model on 2-pollutant Poisson regression by the error variability of PM_2.5_ and NO_2_. Results presented for all scenarios (*N* = 3000 in each row)

CRFs^a^:			PM_2.5_: β_1_ = 5.4^a^			NO_2_: β_2_ = 6^a^		
**Correlation between exposures**	**PM**_**2.5**_**error variability**^**b**^	**NO**_**2**_**error variability**^**b**^	${\hat{\boldsymbol{\beta}}}_{\mathbf{1}}$	**(SE**_**W**_**)/(SE**_**B**_**)**^**c**^	**Bias (%)**^**d**^	${\hat{\boldsymbol{\beta}}}_{\mathbf{2}}$	**(SE**_**W**_**)/(SE**_**B**_**)**^**c**^	**Bias (%)**^**d**^
Low	Very low	Very low	5.44	(1.76)/(4.15)	+ 0.7	5.90	(1.54)/(3.62)	−1.7
		Low	5.49	(1.76)/(4.00)	+ 1.6	5.74	(1.58)/(3.66)	−4.3
		Moderate	6.10	(1.74)/(4.12)	+ 13.0	4.45	(1.71)/(4.02)	−25.8
		High	6.57	(1.73)/(4.08)	+ 21.7	2.85	(1.88)/(4.56)	−52.5
	Low	Very low	5.20	(1.77)/(4.23)	−3.7	6.04	(1.54)/(3.62)	+ 0.7
		Low	5.22	(1.76)/(4.16)	−3.4	5.69	(1.57)/(3.72)	−5.1
		Moderate	5.88	(1.75)/(4.11)	+ 8.9	4.56	(1.70)/(4.17)	−23.9
		High	6.36	(1.74)/(4.05)	+ 17.8	3.00	(1.88)/(4.56)	−50.0
	Moderate	Very low	4.31	(1.81)/(4.30)	−20.2	6.35	(1.53)/(3.58)	+ 5.8
		Low	4.58	(1.80)/(4.29)	−15.3	6.10	(1.56)/(3.78)	+ 1.7
		Moderate	4.94	(1.79)/(4.31)	−8.5	4.88	(1.70)/(4.02)	−18.7
		High	5.42	(1.78)/(4.21)	+ 0.3	3.16	(1.88)/(4.59)	−47.4
	High	Very low	3.46	(1.85)/(4.31)	−36.0	6.64	(1.52)/(3.57)	+ 10.6
		Low	3.72	(1.85)/(4.40)	−31.1	6.37	(1.56)/(3.67)	+ 6.1
		Moderate	4.07	(1.84)/(4.48)	−24.6	5.16	(1.69)/(4.05)	−14.0
		High	4.32	(1.84)/(4.37)	−20.1	3.21	(1.88)/(4.64)	−46.5
Moderate	Very low	Very low	5.36	(1.77)/(4.19)	−0.8	5.99	(1.56)/(3.71)	−0.2
		Low	5.52	(1.77)/(4.34)	+ 2.2	5.59	(1.60)/(3.69)	−6.9
		Moderate	6.12	(1.76)/(4.19)	+ 13.4	4.39	(1.72)/(4.07)	−26.9
		High	6.78	(1.74)/(4.15)	+ 25.5	2.82	(1.89)/(4.56)	−53.0
	Low	Very low	5.06	(1.79)/(4.17)	−6.4	6.06	(1.56)/(3.62)	+ 0.9
		Low	5.15	(1.78)/(4.23)	−4.6	5.73	(1.59)/(3.79)	−4.5
		Moderate	5.87	(1.76)/(4.07)	+ 8.6	4.53	(1.71)/(4.14)	−24.6
		High	6.46	(1.74)/(4.16)	+ 19.6	3.07	(1.89)/(4.54)	−48.9
	Moderate	Very low	4.20	(1.83)/(4.32)	−22.2	6.30	(1.55)/(3.74)	+ 5.1
		Low	4.31	(1.81)/(4.37)	−20.2	6.17	(1.57)/(3.75)	+ 2.9
		Moderate	4.90	(1.80)/(4.26)	−9.3	5.03	(1.71)/(4.13)	−16.2
		High	5.64	(1.79)/(4.30)	+ 4.4	3.11	(1.88)/(4.66)	−48.2
	High	Very low	3.11	(1.86)/(4.42)	−42.3	6.72	(1.54)/(3.60)	+ 12.1
		Low	3.57	(1.85)/(4.40)	−33.9	6.39	(1.56)/(3.67)	+ 6.5
		Moderate	4.17	(1.84)/(4.37)	−22.8	5.26	(1.70)/(4.10)	−12.3
		High	4.55	(1.83)/(4.39)	−15.8	3.17	(1.88)/(4.64)	−47.2
High	Very low	Very low	5.43	(1.78)/(4.15)	+ 0.5	5.89	(1.57)/(3.74)	−1.9
		Low	5.51	(1.78)/(4.21)	−2.1	5.57	(1.60)/(3.86)	−7.3
		Moderate	6.21	(1.76)/(4.09)	+ 15.0	4.30	(1.73)/(4.09)	−28.3
		High	6.81	(1.74)/(4.08)	+ 26.1	2.49	(1.90)/(4.73)	−58.4
	Low	Very low	4.90	(1.80)/(4.24)	−9.3	6.16	(1.57)/(3.70)	+ 2.7
		Low	4.99	(1.82)/(4.27)	−7.5	5.46	(1.62)/(3.91)	−9.0
		Moderate	5.77	(1.78)/(4.19)	+ 6.8	4.36	(1.73)/(4.11)	−27.3
		High	6.35	(1.76)/(4.12)	+ 17.6	2.60	(1.90)/(4.67)	−56.7
	Moderate	Very low	3.97	(1.83)/(4.32)	−26.5	6.27	(1.56)/(3.79)	+ 4.5
		Low	4.17	(1.83)/(4.46)	−22.7	6.07	(1.59)/(3.70)	+ 1.2
		Moderate	4.87	(1.81)/(4.28)	−9.8	4.87	(1.71)/(4.05)	−18.9
		High	5.55	(1.79)/(4.23)	+ 2.8	2.87	(1.89)/(4.73)	−52.1
	High	Very low	3.03	(1.87)/(4.45)	−44.0	6.61	(1.54)/(3.66)	+ 10.1
		Low	3.30	(1.86)/(4.51)	−38.9	6.38	(1.57)/(3.63)	+ 6.4
		Moderate	4.07	(1.85)/(4.42)	−24.7	5.07	(1.70)/(4.12)	−15.5
		High	4.54	(1.84)/(4.41)	−15.8	3.39	(1.88)/(4.60)	−43.4

^a^ Concentration-response functions for the generation of the health outcome

^b^ Moderate error variability as defined in Table 1. Very low = 0.1 x Moderate, Low = 0.5 x Moderate, High = 1.3 x Moderate

^c^ SE_W_: Within-simulations (or model-based) standard error, SE_B_: Between-simulations (or empirical) standard error

^d^ Relative bias = $$ \frac{\left({\hat{\boldsymbol{\beta}}}_{\boldsymbol{\iota}}-{\boldsymbol{\beta}}_{\boldsymbol{\iota}}\right)}{{\boldsymbol{\beta}}_{\boldsymbol{\iota}}} $$

(Classical, Berkson) percentages: (43,57%) for PM_2.5_, (33,67%) for NO_2_

Comparing multi- and single-pollutant model coefficients (β_M_ and β_S_ respectively), Table 4 suggests that, in the absence of ME, using β_S_ may result in a large overestimation of the combined effect of PM_2.5_ and NO_2_. Thus, β_S_, in general, includes effects attributable to both pollutants where both pollutants have a true effect on mortality. For mixture error, regression coefficients increased by 22.5% for PM_2.5_ and 17.7% for NO_2_, when single- instead of multi-pollutant models were fitted. Surprisingly, the combined effect, ${\beta}_{S_1}+{\beta}_{S_2}$, of the mixture error-prone variables was closer to the true combined effect, i.e. 5.4 + 6.0, compared to ${\beta}_{M_1}+{\beta}_{M_2}$. No significant differences were observed between the standard errors of β_M_ and β_S_.

**Table 4:** Summary of the regression coefficients, their standard errors (SE)(x10^-4^) and the percentage decrease from single- to multi-pollutant model estimates for 144,000 simulated datasets on the impact of three error models (classical, Berkson and mixture) on 2-pollutant Poisson regression. Results presented for all scenarios (*N* = 144,000 in each row)

Exposure Model	${\hat{\boldsymbol{\beta}}}_{\mathbf{1}}$	(SE_W_)/(SE_B_)^a^	Bias (%)^b^	Change (%)^c^	${\hat{\boldsymbol{\beta}}}_{\mathbf{2}}$	(SE_W_)/(SE_B_)^a^	Bias (%)^b^	Change (%)^c^
True:								
Multi-Pollutant	5.40	(1.77)/(4.18)	–	+ 32.6	5.99	(1.55)/(3.66)	–	+ 20.5
Single-Pollutant	7.16	(1.71)/(4.06)	–		7.22	(1.50)/(3.55)	–	
Classical:								
Multi-Pollutant	4.65	(1.57)/(3.84)	−13.8	+ 24.7	4.77	(1.35)/(3.40)	−20.5	+ 17.2
Single-Pollutant	5.80	(1.54)/(3.80)	+ 7.3		5.59	(1.32)/(3.38)	−6.9	
Berkson:								
Multi-Pollutant	5.75	(2.41)/(6.32)	+ 6.4	+ 24.7	5.93	(2.17)/(5.49)	−1.2	+ 18.0
Single-Pollutant	7.17	(2.36)/(6.10)	+ 32.8		7.00	(2.13)/(5.37)	+ 16.7	
Mixture:								
Multi-Pollutant	5.03	(1.80)/(4.37)	−6.9	+ 22.5	4.97	(1.68)/(4.25)	−17.1	+ 17.7
Single-Pollutant	6.16	(1.76)/(4.28)	+ 14.1		5.85	(1.62)/(4.21)	−2.4	

^a^ SE_W_: Within-simulations (or model-based) standard error, SE_B_: Between-simulations (or empirical) standard error

^b^ Relative bias = $$ \frac{\left({\hat{\boldsymbol{\beta}}}_{\boldsymbol{\iota}}-{\boldsymbol{\beta}}_{\boldsymbol{\iota}}\right)}{{\boldsymbol{\beta}}_{\boldsymbol{\iota}}} $$

^c^ Percentage change from multi- to single-pollutant estimate = $\frac{\left({\hat{\boldsymbol{\beta}}}_{\boldsymbol{S}}-{\hat{\boldsymbol{\beta}}}_{\boldsymbol{M}}\right)}{{\hat{\boldsymbol{\beta}}}_{\boldsymbol{M}}}$

(Classical, Berkson) percentages: (43,57%) for PM_2.5_, (33,67%) for NO_2_

We performed sensitivity analyses with the Europe, additive, mixture error, core scenario to check which of the simulation inputs are driving ME bias. No difference in the relative bias was observed when we assumed a “true” CRF half or twice the core one. Mortality estimates were attenuated even more when only one pollutant had a true effect on health compared to the case where both had an effect. Interestingly, effect transfer is observed from the pollutant with the true effect to the co-pollutant assumed to have no true effect. When the classical part in the error mixture was increased for both pollutants, the relative bias increased for PM_2.5_ (from 1.3 to 6.0%), while it only decreased slightly for NO_2_ (from 27.4 to 25.4%). Finally, multiplicative error resulted in great underestimation of the exposure-response relationship, i.e. 84.5 and 90.0% for PM_2.5_ and NO_2_ respectively (Table 5).

**Table 5:** Summary of the regression coefficients, their standard errors (SE)(x10^-4^) and relative bias of 48,000 simulated datasets on the impact of mixture error model on 2-pollutant Poisson regression. Results presented for the core scenario (Area: Europe, Error type: Additive-Mixture) and sensitivity analyses (N = 48,000 in each row)

Sensitivity Analysis	CRFs^a^:	PM_2.5_: β_1_ = 5.4^a^			NO_2_: β_2_ = 6^a^		
	Scenario	${\hat{\boldsymbol{\beta}}}_{\mathbf{1}}$	(SE_**W**_)/(SE_**B**_)^**b**^	Bias (%)^**c**^	${\hat{\boldsymbol{\beta}}}_{\mathbf{2}}$	(SE_**W**_)/(SE_**B**_)^**b**^	Bias (%)^**c**^
Main Analysis (Europe-Mixture)		5.33	(1.49)/(3.57)	−1.3	4.36	(2.00)/(5.10)	−27.4
Different “true” CRFs	Low effect CRF^d^	2.66	(1.50)/(3.53)	−1.5	2.19	(2.00)/(4.79)	−27.0
	High effect CRF^e^	10.66	(1.47)/(3.73)	−1.3	8.78	(1.97)/(6.05)	−26.9
	Only PM_2.5_ effect	4.85	(1.50)/(3.57)	−10.2	0.13	(2.02)/(4.76)	–
	Only NO_2_ effect	0.44	(1.50)/(3.55)	–	4.35	(2.02)/(5.05)	−27.5
Mixture error percentages	(Classical,Berkson) PM_2.5_: (55,45%), NO_2_: (45,55%)	5.20	(1.46)/(3.54)	−3.6	4.47	(1.83)/(4.59)	−25.6
	(Classical,Berkson) PM_2.5_: (70,30%), NO_2_: (60,40%)	5.08	(1.42)/(3.45)	−6.0	4.48	(1.70)/(4.26)	−25.4
Error type	Multiplicative	0.83	(0.27)/(1.92)	−84.5	0.61	(0.27)/(1.68)	−90.0

^a^Concentration-response functions for the generation of the health outcome

^b^ SE_W_: Within-simulations (or model-based) standard error, SE_B_: Between-simulations (or empirical) standard error

^c^ Relative bias = $$ \frac{\left({\hat{\boldsymbol{\beta}}}_{\boldsymbol{\iota}}-{\boldsymbol{\beta}}_{\boldsymbol{\iota}}\right)}{{\boldsymbol{\beta}}_{\boldsymbol{\iota}}} $$

^d^ Half the CRF from Mills et al. 2006

^e^ Twice the CRF from Mills et al. 2006

## Discussion

We performed a simulation study to quantify the bias in mortality effect estimates caused by ME in multi-pollutant, time-series models including PM_2.5_ and NO_2_. While the impact of ME can be more easily predicted when single exposures are measured with error, multiple error-prone exposures of any error type (i.e. purely classical, purely Berkson or mixture) can distort the health effect estimates. Our results can be applied to other outcomes and exposures as well.

Mixture error model was found to attenuate the effect of both pollutants, with higher attenuation for NO_2_, the exposure variable measured with more error as found previously [24]. ME also reduced coverage of 95% confidence intervals and statistical power. The largest underestimations of the true effect were in North America for PM_2.5_ (10.3%) and in European studies for NO_2_ (27.4%). Differences by study area were observed because of the different pollutant variability/error variability ratios in these areas and/or potentially other unobserved parameters related to ME. For example, the exposure assessment method used (e.g. measured or modelled concentrations) and the exposure metric (e.g. 24-h mean/max, ambient or personal exposure) may vary by study area. When various values for the correlation between the exposures were assessed, mortality estimates did not change significantly. This is important because correlation between pollutant concentrations is the most common factor discussed in air pollution epidemiological studies as driving unreliability in multi-pollutant models. Day et al. (2004) have reached to similar conclusions for the correlations between the exposures and between the errors except for the case of high correlations (both > 0.8), for a nutritional epidemiology study context [35]. No change was observed in the relative bias with different “true” CRFs, while a small, false positive effect was observed for the pollutant assumed to have no true effect, when both exposures are measured with error. In contrast, when we assumed multiplicative instead of additive error (i.e. a less likely scenario), both pollutant health effect estimates were > 85% biased. Finally, only the PM effect estimate underestimation increased when varying the classical-Berkson ratio of the mixture.

Effect transfer was clearly observed concluding that less precise measurements for one pollutant yield more bias while the co-pollutant effect estimates were closer to the true. This decrease in the bias of the co-pollutant, however, can be regarded as due to the net effect of underestimation due to ME and overestimation due to effect transfer; the latter cancelling out the effect of the former. Szpiro et al. (2011) showed in their simulation study that more accurate exposure predictions do not necessarily improve the health effect estimates [22]. They considered the effects of long-term exposure to air pollution and on comparisons between exposures from correctly specified and misspecified prediction models. However, similar approaches in a multi-pollutant framework have shown that measurement error bias can be severe and correcting for it can strengthen the exposure-response associations [36]. Time-series studies showed that health effect estimates from modelled data are more prone to ME than from measured concentrations [37]. Goldman et al. (2011) reported that spatial error, (only a part of our error decomposition), attenuated the risk ratios from 19 to 31% for primary pollutants (including NO_2_), but only from 2 to 9% for secondary pollutants (PM_2.5_ regarded as such) [30]. These values are close to our overall bias estimates of 17 and 7% respectively, even though their characterisation of NO_2_ and PM_2.5_ as primary and secondary pollutants respectively might be questionable. Similarly, Dionisio et al. *(2016)* fitting two-pollutant time-series models with additive and multiplicative error reported total effect attenuation up to 85% for NO_2_ (close to our estimates for multiplicative error), indicating multi-pollutant model estimates are even more susceptible to ME [38]. Blangiardo et al. (2019) also found, under a Bayesian framework, that NO_2_ effects were considerably biased when error-prone concentrations were used [39]. However, they focused on collinearity in multi-pollutant models without assessing error structures/types.

When misspecified single-pollutant models were fitted, increased effect estimates were observed compared to two-pollutant model coefficients. Interestingly, with mixture error-prone variables, the sum of the single-pollutant model effect estimates was closer to the true combined effect compared with the corresponding sum from multi-pollutant models (due to an accidental cancelling out of overestimation in single-pollutant models from confounding by the co-pollutant and underestimation due to ME). These conclusions hold only when the pollutants are positively correlated. Hence, even if it is accepted that both pollutants have a true causal effect on health [40], should single- or multi-pollutant models be used for quantifying the combined effects of air pollution? Clearly, multi-pollutant models take into account the confounding effects between the pollutants, but if ME is disregarded, biased estimates are produced. This implies that in a “*multi-pollutant air quality management framework*”, ME correction methods should not be neglected [41]. If a correction method cannot be applied, single-pollutant model effects may not be significantly overestimated, but this might only hold under some conditions (e.g. specific correlations between pollutants and/or between errors).

Several previous studies have considered the effects of a mixture of classical and Berkson error in exposures other than air pollution. Mallick et al. (2002) found that the mixture error bias in their relative risks for thyroid disease and radiation fallout ranged from 3.2 to 42.7% [15]. Tapsoba et al. (2019) studying medications in HIV patients report biases from 0 to 22% depending on the correction method [33]. These values are close to our findings, as is their assumed percentage of Berkson error in the exposure that lies between 20 and 80%. In contrast, Deffner et al. (2018), examining the effects of ultra-fine particles on heart rate, reported that mixture error had little impact on their results [17]. This, however, may be due to their error definitions: they assumed that total personal measurements include only classical error, measurements from fixed sites only Berkson.

This simulation study has some limitations. Firstly, several of our assumptions increased the uncertainty of our estimates. For example, we used pooled estimates for the error structures across wide study areas (e.g. cities within our areas may have different concentrations), and approximations for the correlation between exposures and between errors. However, to the best of our knowledge, no study has attempted to quantify the important error variables in specific areas, apart from Dionisio et al. (2014) in Atlanta (using a different, more spatial framework than our study) [23]. We used more generalisable inputs provided from previously published work [24]. In addition, equal exposure misclassification across days was assumed, increasing the uncertainty about the relationship between sources of spatial and temporal variations. We could not investigate the dependency or correlation between daily exposures and how this might interact with the spatial variation. Moreover, we assumed a constant percent error model additive on the logarithmic scale which implies that at low exposures, error is also low, and vice versa. Error may be higher at high exposures, but it might not drop substantially at lower concentrations. In future work, we plan to use real data from panel studies to identify the gaps in our information and describe properly the error structures of the air pollutants.

This study discusses PM_2.5_ and NO_2_ and how to separate their effects on health. There is debate over whether NO_2_ is acting as an indicator for other traffic pollutants. To the extent that these other pollutants have similar spatial error characteristics and some, e.g. CO, ultrafine particles, may also have greater infiltration indoors than PM_2.5_, our findings regarding effect transfer in multi-pollutant models with PM_2.5_ will also apply. Moreover, our work could not quantify the error structures of ozone so could not assess its exposure misclassification bias. This work was on two pollutants that are generally positively correlated. Ozone, due to its formation and characteristics, can be negatively correlated with PM_2.5_ and NO_2_, and this may change our conclusions. However, the correlations are not expected to be very high, and according to this and previous studies, the effect of pollutant correlations on ME bias is not expected to be substantial [35]. We are currently working on this, using previously analysed raw data from a panel study on schoolchildren [42]. We will also assess the impact of ME on the shape of the exposure-response curve. This work did not find any significant distortions for the shape of the CRFs for the health effects of short-term exposures when the error is additive, but the effects of multiplicative error might be more profound especially for the identification of the long-term effects of air pollution, for which the CRFs are used in cost-benefit analyses [43]. We addressed effect estimates from short-term exposures. Future work should address the multi-pollutant model estimates for long-term exposures which may be more biased, as indicated in studies assessing single-pollutant exposures [31, 32]. Finally, measurement error correction is an important aspect which becomes more complex if we consider expanding the findings from exposure to air pollution to the whole exposome, i.e. the totality of environmental exposures throughout a lifetime. In such studies, variable selection methods are usually used, which rely on empirical data and may create false positive and false negative selections in the presence of ME [44].

In summary, this study quantified the effects of exposure measurement error on multi-pollutant, time-series model estimates. Using simulations, under an extensive range of scenarios, we showed that non-trivial underestimation in health effect estimates can result from measurement error, especially for NO_2_, which was found to be more prone to error, but for PM_2.5_ as well. We recommend that ME should be considered in every epidemiological analysis assessing exposures prone to large ME, and that studies of personal exposure should provide information on relevant error parameters, such as correlation between errors and error variability, in order to better understand the correct error structures of the pollutants. It is important that correct health effect estimates should be derived in order, not only to separate the independent effects of air pollutants, but also to correctly quantify the health impacts of air pollution, inform interpretation and recommend future approaches for policy making.

## Supplementary information

**Additional file 1.**

## Data Availability

The datasets used and analysed during the current study are available from the corresponding author on reasonable request. This work was produced using simulated data.
