# Supplementary material for: Quantifying the short-term effects of air pollution on health in the presence of exposure measurement error: a simulation study of multi-pollutant model results
Source: Environ Health. 2021 Aug 24;20:94. doi: 10.1186/s12940-021-00757-4 (PMC8385952; doi:10.1186/s12940-021-00757-4)
Supplement: Supplementary file 1 — Additional file 1. [file 12940_2021_757_MOESM1_ESM.docx]

**Quantifying the short-term effects of air pollution on health in the presence of exposure measurement error: A simulation study of multi-pollutant model results. – Supplementary Material**

*
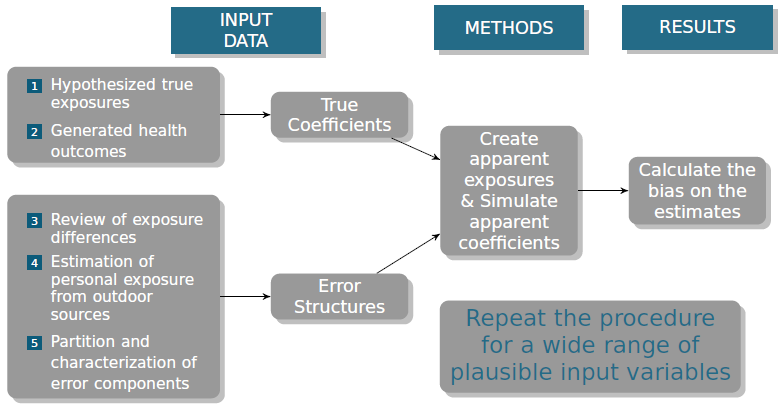
Simulation process:*

Figure S1 - Flow diagram of the simulation procedure followed in this work.

*Generating Berkson error:*

The algorithmic procedure to create exposures of purely Berkson type of error was the following:

1. Estimate the variance of the error-prone variables, $\sigma_{C}^{2}$. We have

$$\sigma_{A}^{2}=\sigma_{C}^{2}+\sigma_{\delta}^{2}\Longrightarrow\sigma_{C}^{2}=\sigma_{A}^{2}-\sigma_{\delta}^{2}$$

If $\sigma_{A}^{2}<\sigma_{\delta}^{2}$, we assigned a percentage of $\sigma_{C}^{2}$ from the previous error scenario where $\sigma_{A}^{2}\geq\sigma_{\delta}^{2}$.

1. From a bivariate normal distribution generate$\left[ \begin{matrix} \delta_{1}^{*} \\ \delta_{2}^{*} \end{matrix} \right]$ with mean $\mu_{\delta_{\iota}^{*}}=0$, variance $\sigma_{\delta_{\iota}^{*}}=\frac{\sigma_{C}^{2}\cdot\sigma_{\delta}^{2}}{\sigma_{C}^{2}+\sigma_{\delta}^{2}}$ and covariance:

$$Cov\left( \delta_{1}^{*},\delta_{2}^{*} \right)=Cov[C_{1}-\left( \mu_{A_{1}}+\left( A_{1}-\mu_{A_{1}} \right)\cdot\lambda_{1} \right),C_{2}-\left( \mu_{A_{2}}+\left( A_{2}-\mu_{A_{2}} \right)\cdot\lambda_{2} \right) ]$$

and with some basic algebraic calculations we have:

$Cov\left( \delta_{1}^{*},\delta_{2}^{*} \right)=Cov\left( C_{1},C_{2} \right)+\lambda_{1}\cdot\lambda_{2}\cdot Cov\left( A_{1},A_{2} \right)-\lambda_{1}\cdot Cov\left( A_{1},C_{2} \right)-\lambda_{2}\cdot Cov\left( A_{2},C_{1} \right)$.

The covariances above were quantified from the relationships between T_i_ and C_i_, as we couldn't find any study reporting associations between errors defined in the same way as in our study.

Finally, an error prone variable of entirely Berkson error was created based on:

$C=\mu_{A}+\left( A-\mu_{A} \right)\cdot\frac{\sigma_{C}^{2}}{\sigma_{C}^{2}+\sigma_{\delta}^{2}}+\delta^{*}$.

*Generating mixture error:*

The algorithmic procedure for the generation of mixture error type is described below:

1. Let $\sigma_{\delta_{tot}}^{2}$ be the total error variance as quantified in Evangelopoulos *et al.* (2020) and used as input in our scenarios.(1) Then if we assume that p% of the total error is of Berkson and (1-p)% is of classical type, we have:

$\sigma_{\delta_{b}}^{2}=p\cdot\sigma_{\delta_{tot}}^{2}$ and $\sigma_{\delta_{c}}^{2}=(1-p)\cdot\sigma_{\delta_{tot}}^{2}$.$Equation S1$

1. Based on equations 1 and 2 (main text) and the error properties, the error-free and error-prone exposure variances are quantified as follows:

$\sigma_{A}^{2}=\sigma_{L}^{2}+\sigma_{\delta_{b}}^{2}$ and $\sigma_{C}^{2}=\sigma_{L}^{2}+\sigma_{\delta_{c}}^{2}$.

Thus, using the input values for $\sigma_{A}^{2}$, $\sigma_{\delta_{tot}}^{2}$, we managed to estimate $\sigma_{L}^{2}$ and $\sigma_{C}^{2}$.

1. The intermediate variable **L** is created as a Berkson error-prone surrogate of the assumed true exposures, based on equation 3 (main text) and the algorithm described in the previous paragraph:

$$L=\mu_{A}+\left( A-\mu_{A} \right)\cdot\frac{\sigma_{L}^{2}}{\sigma_{L}^{2}+\sigma_{\delta_{b}}^{2}}+\delta^{*}$$

where $\delta_{b}^{*}\sim N(0,\frac{\sigma_{L}^{2}\cdot\sigma_{\delta_{b}}^{2}}{\sigma_{L}^{2}+\sigma_{\delta_{b}}^{2}})$.

1. The mixture error-prone exposures are generated as a classical error-prone exposure of the intermediate variable **L**. More specifically, we have $C=L+\delta_{c}$, where **L** was generated in step 3 and δ_c_ was quantified from equation S1, $\delta_{c}\sim N(\boldsymbol{0}, \boldsymbol{\Sigma})$. The covariance between the errors was calculated as described in the classical error paragraph. The Higham (2002) algorithm was applied when the correlation matrix set in the iterative procedure was not positive (semi-)definite.(2) This algorithm, used in various scientific fields, computes the nearest positive definite matrix to a correlation matrix which is used as input and fails to be semi-definite.(2, 3)

*Exposure data in the simulations:*


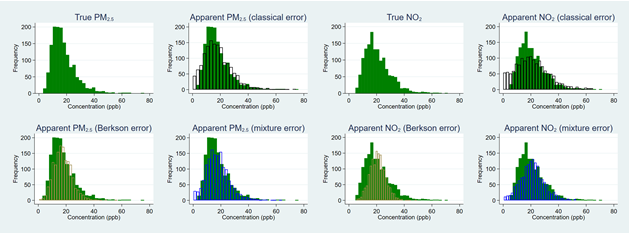
Figure S2 presents histograms of the assumed error-free and error-prone PM_2.5_ and NO_2_ concentrations from one of the 144,000 generated datasets. As expected, all the pollutant-specific exposure variables have similar mean values: around 18μg/m^3^ for PM_2.5_ and around 21ppb for NO_2_ (see Table 1 in the main text). The classical error-prone exposure is more variant than the assumed true exposure, while the latter is more variant than the Berkson error-prone exposure. The mixture case combines elements of both.

Figure S2 - Histograms of the error-free (green) and error-prone (transparent) daily mean exposures from one randomly selected scenario.

References

1. Evangelopoulos D, Katsouyanni K, Keogh RH, Samoli E, Schwartz J, Barratt B, et al. PM2. 5 and NO2 exposure errors using proxy measures, including derived personal exposure from outdoor sources: A systematic review and meta-analysis. Environment International. 2020;137:105500.

2. Higham NJ. Computing the nearest correlation matrix—a problem from finance. IMA journal of Numerical Analysis. 2002;22(3):329-43.

3. Demirtas H, Hedeker D, Mermelstein RJ. Simulation of massive public health data by power polynomials. Statistics in Medicine. 2012;31(27):3337-46.
